# For Flyless Cities and Towns

**Published:** 1914-05

**Authors:** Josephine Daniel


### For Flyless Cities and Towns.

The country is going to have to depend on the women to create the sentiment that will result in the extermination of the flies. Most people realize in a general way that the fly is a pest, some know that it spreads disease, but most of them seem to think that they can continue to dodge the flies and avoid "catching" anything themselves. It is surprising to learn the number of epidemics and diseases that have been traced by scientists directly to fly-spread germs. The fly has been proven to be a greater menace than poisonous snakes, mankilling beasts, or even the bullets of war.

They are to be found in myriads in the marketplaces, spreading germs everywhere with their fifth-laden feet, tracking over the meats, the bread, the vegetables and the cooked foods. Many of the grocery stores are unscreened, or, if screened, have the flies snugly gathered within their doors to roam at will over the provisions sent out to their customers. The delivery wagons are seldom covered, and the flies feed at pleasure on the poor horse that pulls the wagon, on the manure heap in the street, and on the provisions brought to your door. Many of these wagons are kept over night in filthy barns close by the horses and cows, and are used as roosting places for swarms of flies until they are needed for the groceries. They are never cleaned, except as the groceries and vegetables serve as mops.

Little children in screenless homes, or from better homes when at play, are especially plagued with these pests that imperil their lives all the while.

It is therefore plainly the duty of the housekeepers and the mothers of this country to get active in the campaign against these perilous pests. Spasmodic fly-swatting will never exterminate the flies. Every fly that can be found should be promptly swatted, houses should be well screened, and every precaution against them should be taken in the home, but to exterminate the fly there must be far-reaching anti-fly campaigns conducted in every city, town and village. Stable refuse is the most prolific breeding place for flies, and unclean streets and alleys filled with decaying garbage and old rags and scraps are choice fly hatcheries. A flyless town must be a clean town, and every part of it must be clean.

Some of the largest places in the country have practically rid themselves of flies, and with the same vigilant methods this can be accomplished anywhere. See that strict city ordinances are passed giving authority to make short shrift of the fly-breeders. Let the business men know that they must co-operate or they will lose their business. Get local physicians to write articles showing how public life is constantly imperiled by flies. Secure the help of the editors, who are always ready to join in everything for the public good, and create a sentiment for cleanliness and healthfulness that will make yours a flyless place. But remember, that, however, much you may entrap, or swat, or poison, you are but temporizing with the fly family so long as the breeding places are undisturbed.—*Josephine Daniel.*

